# The Positive Effects of Training and Time-Restricted Eating in Gut Microbiota Biodiversity in Patients with Multiple Myeloma

**DOI:** 10.3390/nu17010061

**Published:** 2024-12-27

**Authors:** Olga Czerwińska-Ledwig, Alicja Nowak-Zaleska, Małgorzata Żychowska, Katarzyna Meyza, Tomasz Pałka, Adrianna Dzidek, Agata Szlachetka, Artur Jurczyszyn, Anna Piotrowska

**Affiliations:** 1 Institute for Basic Sciences, Faculty of Physiotherapy, University of Physical Education, 31-571 Krakow, Polandadrianna.dzidek@doctoral.awf.krakow.pl (A.D.); 2 Department of Biological Foundations of Physical Culture, Kazimierz Wielki University, 85-091 Bydgoszcz, Poland; alicja.nowak-zaleska@ukw.edu.pl (A.N.-Z.); malgorzata.zychowska@ukw.edu.pl (M.Ż.); 3 Department of Genetics, Faculty of Biological Sciences, Kazimierz Wielki University, Powstańców Wielkopolskich 10, 85-090 Bydgoszcz, Poland; 4 Department of Physiology and Biochemistry, Faculty of Physical Education and Sport, University of Physical Education, 31-571 Krakow, Poland; tomasz.palka@awf.krakow.pl; 5 Doctoral School of Physical Culture Science, University of Physical Education, 31-571 Krakow, Poland; 6 Faculty of Medicine and Health Sciences, Tarnów Academy, 33-100 Tarnów, Poland; a_szlachetka@anstar.edu.pl; 7 Plasma Cell Dyscrasia Center, Department of Hematology, Faculty of Medicine, Jagiellonian University Medical College, 31-501 Krakow, Poland; mmjurczy@cyf-kr.edu.pl

**Keywords:** physical activity, Nordic walking, multiple myeloma, time-restricted eating, intestinal microbiota, hematological malignancies, gut microbiome

## Abstract

Background: The physical activity of different groups of individuals results in the rearrangement of microbiota composition toward a symbiotic microbiota profile. This applies to both healthy and diseased individuals. Multiple myeloma (MM), one of the more common hematological malignancies, predominantly affects older adults. Identifying an appropriate form of physical activity for this patient group remains a challenge. The aim of this study was to investigate the impact of a 6-week Nordic walking (NW) training program combined with a 10/14 time-restricted eating regimen on the gut microbiota composition of multiple myeloma patients. Methods: This study included healthy individuals as the control group (n = 16; mean age: 62.19 ± 5.4) and patients with multiple myeloma in remission (MM group; n = 16; mean age: 65.00 ± 5.13; mean disease duration: 57 months). The training intervention was applied to the patient group and consisted of three moderate-intensity sessions per week, individually tailored to the estimated physical capacity of each participant. The taxonomic composition was determined via 16S rRNA sequencing (V3–V9 regions). The microbiota composition was compared between the patient group and the control group. Results: The alpha and beta diversity metrics for species and genus levels differed significantly between the control and patient groups before the implementation of the NW program. In contrast, no differences were observed between the control and patient groups after the training cycle, indicating that the patients’ microbiota changed toward the pattern of the control group. This is confirmed by the lowest values of average dissimilarity between the MMB groups and the control at all taxonomic levels, as well as the highest one between the control group and the MMA patient group. The gut microbiota of the patients was predominantly represented by the phyla *Firmicutes*, *Actinobacteria*, *Verrucomicrobia*, *Proteobacteria*, and *Bacteroidetes*. Conclusions: The training, combined with time-restricted eating, stimulated an increase in the biodiversity and taxonomic rearrangement of the gut microbiota species.

## 1. Introduction

In recent years, interest in the taxonomic composition of microorganisms (microbiota) and their physiological potential (enzymes, toxins, metabolic products, etc.) as determined by their genome (microbiome) has increased. Thanks to modern genetic material sequencing technologies [1,2,3], it is possible to gain detailed knowledge about the composition of the gut microbiota. Next-generation sequencing (NGS) allows for the parallel sequencing of thousands of DNA samples, which significantly shortens sequencing time, and, when combined with nanopore technology, provides information about the nucleotide composition of long sequences, the gene content in the analyzed pool, and thus knowledge about the microbiome or microbiota when taxonomic composition is being studied. In studies on species composition, bacterial taxonomy most commonly uses 16S rRNA gene sequencing [4,5,6]. At least 800 different bacterial taxa at the species level have been identified from the feces of healthy individuals [7,8,9]. The stability of the microbiota composition is low, and the microbiota undergoes constant changes in response to external factors such as nutritional status, environmental factors, lifestyle, health condition, or diseases [4].

A review of the literature on physical activity and its impact on the rearrangement of microbiota composition indicates an increase or emergence of populations of microorganisms considered to have a positive impact on human health (so-called symbiotic microbiota) [4,5,6,10]. The effects of physical activity on microbiota composition have been described for various forms of physical activity or physical training. Studies conducted by the Bressa team revealed a change in the microbiota profile in healthy individuals who engage in low-dose but continuous physical activity, leading to an increase in the abundance of bacteria considered beneficial for health (e.g., *Bifidobacterium* spp., *Roseburia hominis*, *Akkermansia muciniphila*, *Faecalibacterium prausnitzii*) [5]. A review of studies on the impact of physical exercise or physical activity in both healthy individuals and patients (chronic diseases—such as obesity, metabolic syndrome, diabetes, atherosclerosis, liver dysfunction, and inflammatory bowel disease (IBD)) on gut microbiota indicates a rearrangement of the microbiota profile in both populations. The direction and scope of changes are determined by the health status and the intensity and duration of physical exercise [6]. The evaluation of data presented by Humińska-Lisowska et al. [10] shows that changes in microbiota in the group of healthy, fit, and physically active individuals—strength and endurance athletes, as well as the control group—indicate similar microbiota features in all participants, confirming mutual positive interactions. However, in the group with a high volume of training or endurance exercises, an increase in populations of carbohydrate-degrading bacteria was observed. Authors noted that microbiota diversity correlates with VO_2_max. They also found that performance parameters, most strongly VO_2_max, are positively correlated with species such as *Bifidobacterium longum*, *Bifidobacterium adolescentis*, and *Prevotella*. A negative correlation of VO_2_max was estimated for *Bacteroides*. Endurance loads (endurance sports and associated training) show a correlation with the presence of butyrate producers among short-chain fatty acids (*Blautia wexlerae*, *Eubacterium rectale*, *Intestinimonas timonensis*), which is associated with the power output of physically active individuals.

Studies already performed on the gut microbiota in patients with multiple myeloma have indicated a correlation between its composition and the immunological specificity of the gut environment [11,12,13,14], as well as a distinct microbiota composition compared to healthy individuals [15,16]. In a group of patients with multiple myeloma, Korde et al. observed a positive effect of physical activity on the patient’s health during successive cycles of treatment [17]. Patients in this study were continuously monitored; their physical activity level was assessed based on the number of steps taken per day. Prior to the introduction of the therapeutic cycle, activity was at the level of 4818 steps in the cohort under 65 years of age and 6000 steps/day in the older cohort. After the last studied cycle, activity ranged from 6300 to 7300 steps/day, which, as the authors noted, positively influenced the treatment process. Activity trends were associated with improvements in physical functioning and overall health status. A negative correlation was also observed between physical activity levels and disease burden symptom scores [17]. Surprisingly, older patients showed greater increases in activity (+260 steps/24 h per cycle) compared to patients in the >65-year-old cohort (+116 steps/24 h per cycle).

Another significant factor affecting the gut microbiota is time-restricted eating (TRE) [18]. It is already known that the TRE/time-restricted feeding (TRF) approach is essential for the targeted utilization of nutrients and the metabolism of the host’s nutritional status through the modulation of the gut microbiota and circadian rhythms. This approach has shown a widespread effect in restoring gut microbiota dysbiosis. TRE/TRF may also contribute to the prevention of metabolic diseases by modulating the Clock-Bmal1 pathway, synchronizing hormonal signals, regulating the Sirt1 pathway, inhibiting mTOR signaling, and modulating nutrient-sensing receptors associated with the gut microbiota. This type of diet has already been studied in groups of patients undergoing Nordic walking (NW) training, indicating beneficial effects [19,20]. Furthermore, the potential for combining training interventions in the form of NW with TRE in patients with multiple myeloma has already been suggested [21].

The intestinal environment of people with obesity or MM creates conditions for microbial growth to a limited extent; hence, their microbiota is characterized by reduced diversity. Studies by various authors indicate that the diversity of the microbiota is stimulated by physical activity in different groups of people. Considering the impact of physical activity on the rearrangement of the gut microbiota profile, we aimed to determine whether a 6-week Nordic walking training combined with a dietary change involving the introduction of TRE (10/14) would induce positive changes in the gut microbiota of patients with multiple myeloma. Referring to the literature, we hypothesized that the microbiota of healthy individuals would differ from that of patients, with a lower diversity in the patient group compared to healthy individuals. Similarly, the microbiota of patients before and after the training cycle would differ in terms of species composition and abundance. We also hypothesized that the diversity of the microbiota in patients would be stimulated with the proposed dietary and training interventions.

## 2. Materials and Methods

### 2.1. Characteristics of the Patient Group and Healthy Controls

The study protocol received a positive opinion from the Bioethics Committee. Each participant in the project was informed about the purpose and methods of the study, and written consent was provided to participate. Participants were given the option to withdraw from the study at any stage without providing a reason.

The study group consisted of 20 patients in the plateau phase of multiple myeloma, recruited from the Department of Hematology at the Jagiellonian University Collegium Medicum in Kraków. Patients were selected for participation in the project by the attending physician based on a list of inclusion and exclusion criteria. The inclusion criteria for this group were as follows: multiple myeloma in the plateau phase, without cytostatic treatment; bisphosphonate therapy was permitted; generally good health status with no contraindications for outdoor physical activity, specifically Nordic walking training; supplementation of vitamin D and calcium according to standard protocols. The exclusion criteria included the following: significant liver and kidney damage, acute respiratory or other infections, any other malignant cancer, recent fall resulting in injury, and antibiotic therapy in the last 3 months.

**Figure 1:**
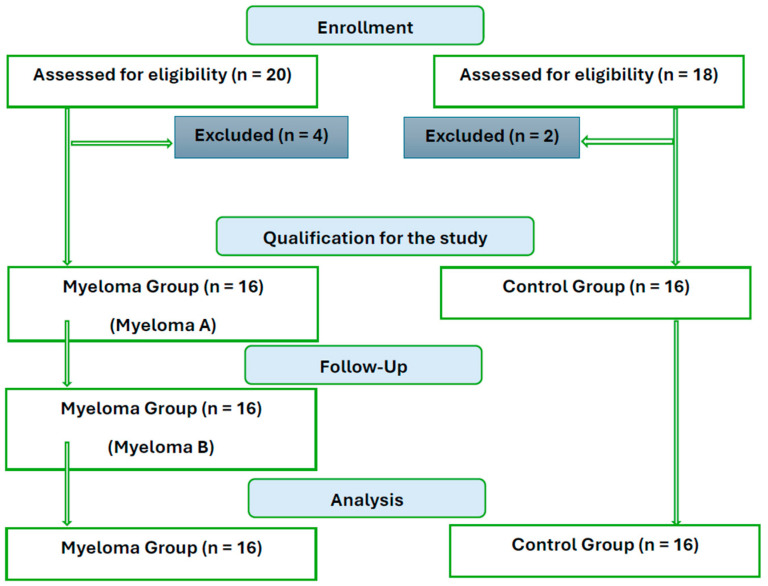
Patient diagram flow.

The control group was created by selecting healthy volunteers of a similar age from the general population who met the following criteria: no antibiotic therapy in the 3 months prior to the study, no infectious diseases or acute inflammatory conditions, no uncontrolled chronic diseases, diabetes, or contraindications to physical activity. Out of the selected group of 20 participants, 2 withdrew without providing a reason. From the study group, 4 individuals were excluded due to the use of antibiotic therapy less than 3 months prior to joining the project. Figure 1 shows the patient flow diagram. The basic characteristics of the project participants are presented in Table 1.

### 2.2. Nordic Walking Training Protocol

Nordic walking (NW) training was conducted by an experienced instructor, skilled in working with seniors and oncology patients, outdoors during the spring and summer period. The intensity of the training was planned at 60–70% of HRmax, individually calculated using the Nes formula [22].

Blood pressure measurements were taken before each session. The intensity of the training was monitored using sport testers (M400, Polar, Kempele, Finland), with user data input, including permissible heart rate levels. If these levels were exceeded, the device emitted an audible signal, and the instructor adjusted the intensity of the participant’s exercises to ensure that the heart rate did not exceed the designated range for moderate-intensity effort.

The planned 6-week health training cycle consisted of 18 training sessions, each lasting approximately 60 min, conducted three times a week in the morning. In successive training sessions (starting from session 4), the duration of each session was gradually increased (up to 45 min), as well as the distance covered by the participants, while maintaining proper Nordic walking technique.

The training session is described as follows: (1) Warm-up: 10 min (Borg scale 6–8: light effort). It included general exercises for the upper and lower limbs and the trunk, as well as stretching exercises adapted to the needs of the group. (2) Main part: up to 45 min, with intensity set at 60–70% HRmax (Borg scale 10–12: moderate effort, allowing for easy conversation). This part consisted of exercises with poles aimed at learning and reinforcing the Nordic walking technique. The next stage was walking with poles, maintaining proper Nordic walking technique. With each successive training session, the walking time was gradually increased until reaching a maximum of 45 min. During this part, patients strictly adhered to recommendations regarding the maximum heart rate they could achieve during training. (3) Cool-down: 5 min (Borg scale 6–8: light effort). Patients performed stretching exercises for different body parts.

### 2.3. Time-Restricted Eating

The dietary modification in the form of fasting was implemented daily. Participants were instructed to refrain from consuming food for 14 h each day within individually chosen time windows. The eating window lasted for 10 h. No other dietary restrictions were specified. Patients were advised not to change their eating habits and to continue with their self-composed diet. To ensure adherence to the dietary regimen, the time-restricted Eating (TRE) was monitored by the trainer during each training session.

### 2.4. Stool Collection and DNA Extraction and Sequencing

Participants were provided with sterile plastic containers for stool sample collection. The samples were collected just before the start of the training and on the last day of the physical sessions. The samples were stored at −80 °C until DNA isolation. DNA isolation was performed using a Fecal DNA Extraction Kit (IBI Scientific, Dubuque, IA, USA), with an additional custom modification. A sample (approximately 200 mg) was suspended in 800 µL of ST1 buffer, vortexed for 20–30 s, and then incubated for 5 min at 70 °C. In the next step, the sample was vortexed at maximum speed in a horizontal position for 5 min at room temperature, followed by centrifugation for 4 min at room temperature (6000× *g*). The supernatant (550 µL) was transferred to a new 1.5 mL tube and centrifuged again for 1 min (8000× *g*). Next, 500 µL of the supernatant was transferred to a new 1.5 mL tube, 150 µL of ST2 buffer was added, vortexed for 5 s, and incubated at 0–4 °C for 5 min. After cooling, the sample was centrifuged at 15,000× *g* for 5 min at room temperature. The supernatant (500 µL) was transferred to the inhibitor removal column and centrifuged for 1 min at 16,000× *g*. To the sample, 800 µL of ST3 buffer was added, mixed thoroughly for 10–15 s, and 700 µL was transferred to the GD column. The sample was centrifuged at 16,000× *g* for 2 min, and the GD column was transferred to a 2 mL tube. Then, 400 µL of ST3 buffer was applied to the GD column and centrifuged at 16,000× *g* for 30 s. The GD column was washed twice with 600 µL of Wash Buffer, centrifuged at 16,000× *g* for 30 s. The GD column was transferred to a 2 mL tube and centrifuged at 16,000× *g* for 3 min. DNA was eluted using 55 µL of Elution Buffer; after incubation for 6 min at room temperature, the sample was centrifuged at 16,000× *g* for 3 min.

### 2.5. Sequencing and Bioinformatics

The V3–V9 regions of the 16S rRNA gene were amplified using the universal primers 337F and 1391R. The resulting amplicons were subjected to sequencing using nanopore technology with the native barcoding 1D protocol. Sequencing was performed on R9.4.1 flowcells with default settings. The taxonomic classification of the obtained reads was carried out using the ublast algorithm (genXone, Złotniki, Poland). This classification is based on comparing read sequences with a database (NCBI). If the genomic sequence of the searched organism had not been previously deposited in the database (NCBI), it is not displayed in the classification results. Reads derived from organisms not present in the database were assigned to another, the most similar sequence, e.g., a sequence representing a higher taxonomic level, or discarded. A taxonomic classification table was created in a format compatible with the interactive Pavian taxonomic classification browser (available online at https://fbreitwieser.shinyapps.io/pavian, accessed on 1 June 2024). The sample diversity index was used, separated by the cutoff level as the minimum number of reads for a taxon (0: no cutoff, 5, 15, 30, 50), as well as a method for index creation, the Simpson index. Cluster analysis was performed using ClustVis (http://biit.cs.ut.ee/clustvis, accessed on 1 June 2024).

### 2.6. Statistical Analysis

In the statistical analysis, alpha- and beta-diversity indices were estimated. Three alpha-diversity indices were calculated: Shannon diversity index, Fisher’s index, and Simpson’s index (Simpson 1-D). Beta-diversity was characterized using the Bray–Curtis dissimilarity metric. The ANOSIM and PerMANOVA tests (Permutational MANOVA, also known as NPMANOVA) were used for the comparison of sample groups, as well as ANOVA tests (including the Mann–Whitney and Kruskal–Wallis tests). A *p*-value < 0.05 was considered statistically significant. Additionally, to determine which taxa are primarily responsible for the observed differences between sample groups, the SIMPER tool [23] was used. The Bray–Curtis metric was used as the data matrix in the UMAP analysis to illustrate the mutual similarities and differences between microbiome samples [24]. The statistics were calculated using PAST4.17 software (Hammer 2001) [25].

## 3. Results

### Microbiota Composition Diversity

**Figure 2:**
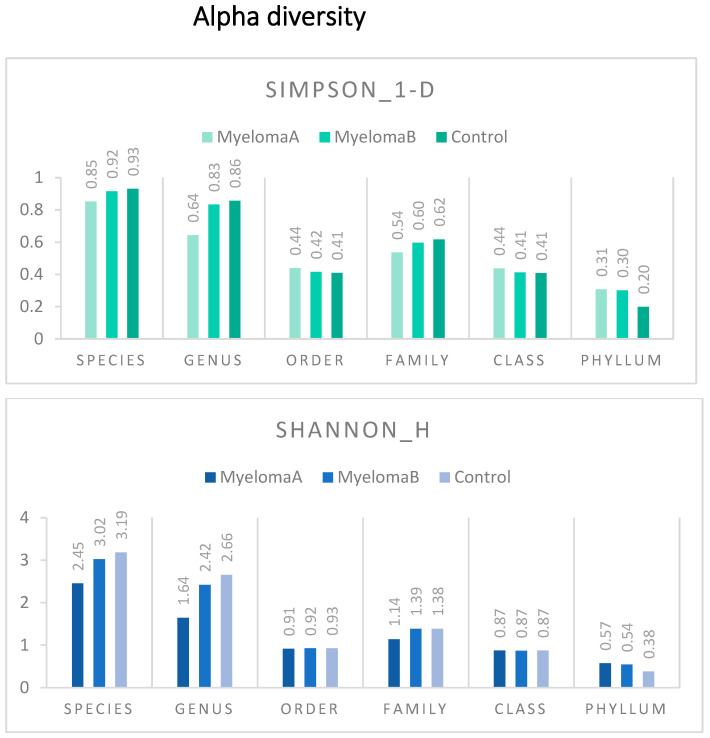

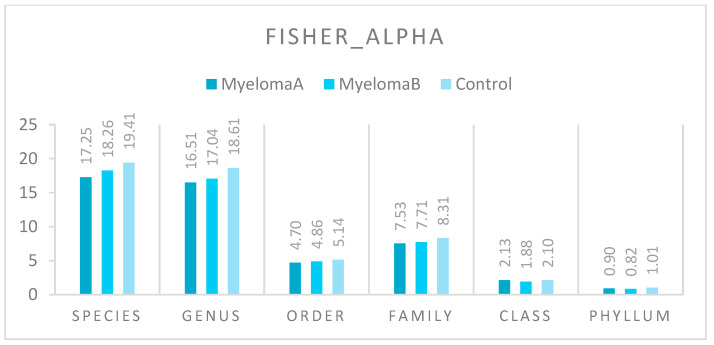
Graph for the studied alpha-diversity metrics: Simpson’s Index (1-D), Shannon Index, and Fisher-alpha parameter (median) considering the taxonomic levels of the microbiota and the division of the study sample into groups (MyelomaA—group of patients before starting the NW program; MyelomaB—group of patients after completing the Nordic walking program; control).

The global assessment of alpha-diversity in the studied sample, as well as within the control group (CG), the group of patients with multiple myeloma (MM) before starting the Nordic walking training program (MMA), and that after completing the Nordic walking program (MMB) indicates greater species diversity along with a decrease in taxonomic rank (the highest for species: Species; the lowest for phylum: Phylum). Attention is drawn to the lower level of the alpha-diversity (Simpson 1-D and Shannon H) in the control group compared to the patient group (MMA, MMB) at the phylum taxonomic rank. This trend is reversed when considering the alpha-diversity structure at the genus (Genus) and species (Species) levels, where higher diversity coefficients are observed in the control group and lower in the patient group. Interestingly, the diversity coefficients in the patient group after completing the training cycle approached those in the control group at the Species, Genus, and Family ranks. Higher values of the estimated indices indicate greater species diversity and their more or less even distribution (Figure 2). Moreover, statistically significant differences were observed between the studied groups for all estimated alpha-diversity indices at the species and genus levels. Statistically significant differences were also found when comparing the microbiota of the control group and the patient group before starting the NW training for the aforementioned taxonomic ranks (Table 2).

**Figure 3:**
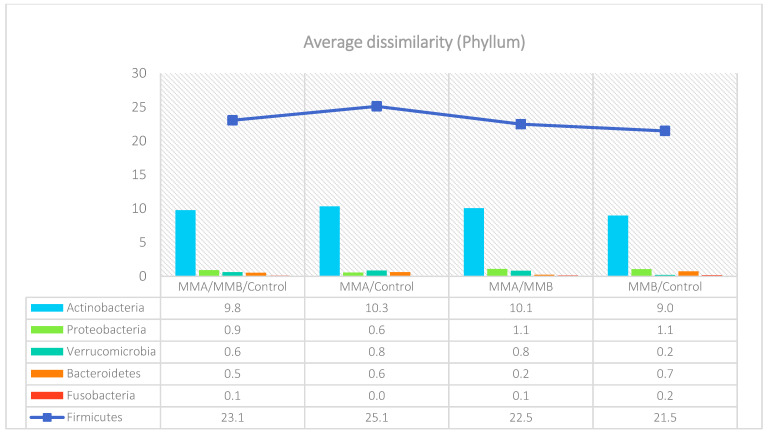
Mean divergence between the study groups (MMA—group of patients before starting the NW program; MMB—group of patients after completing the Nordic walking program at the phylum level.

**Figure 4:**
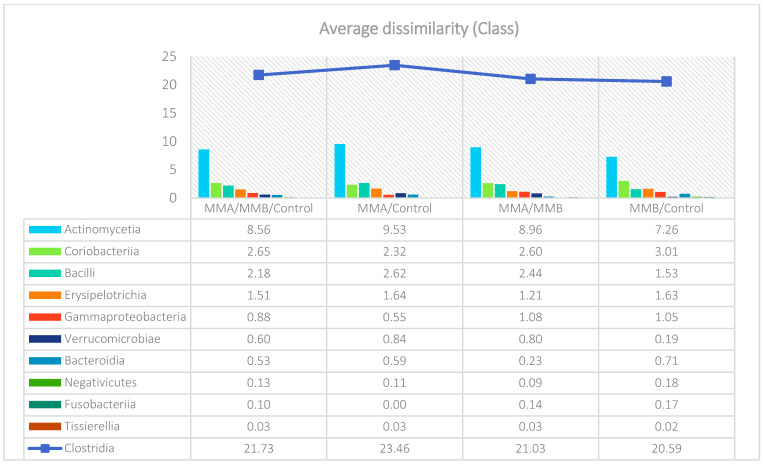
Mean divergence between the study groups (MMA—group of patients before starting the NW program; MMB—group of patients after completing the Nordic walking program) at the class level.

**Figure 5:**
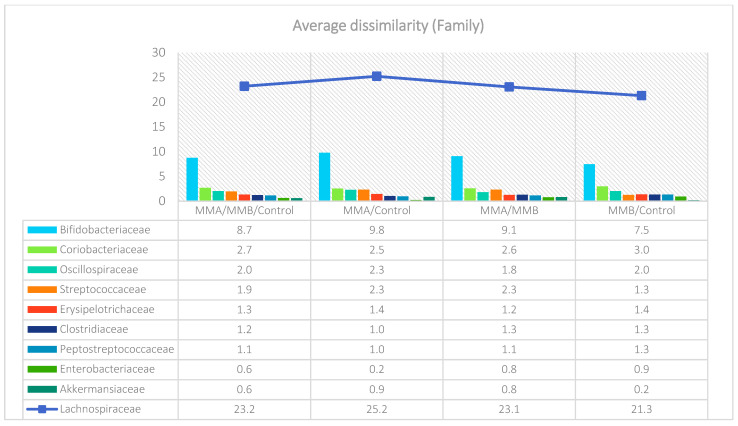
Mean divergence between the study groups (MMA—group of patients before starting the NW program; MMB—group of patients after completing the Nordic walking program) at the family level.

**Figure 6:**
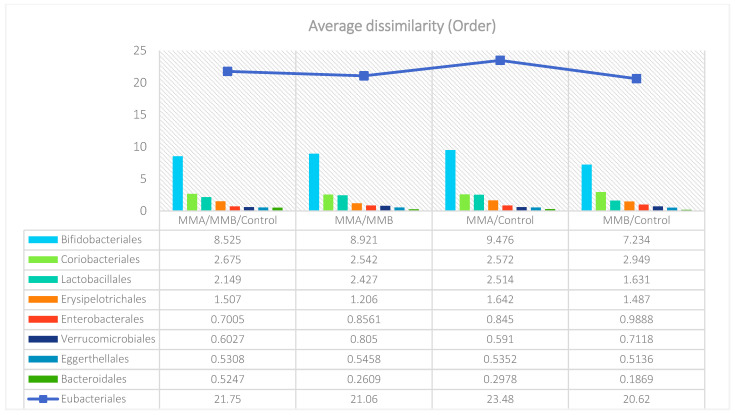
Mean divergence between the study groups (MMA—group of patients before starting the NW program; MMB—group of patients after completing the Nordic walking program) at the order level.

**Figure 7:**
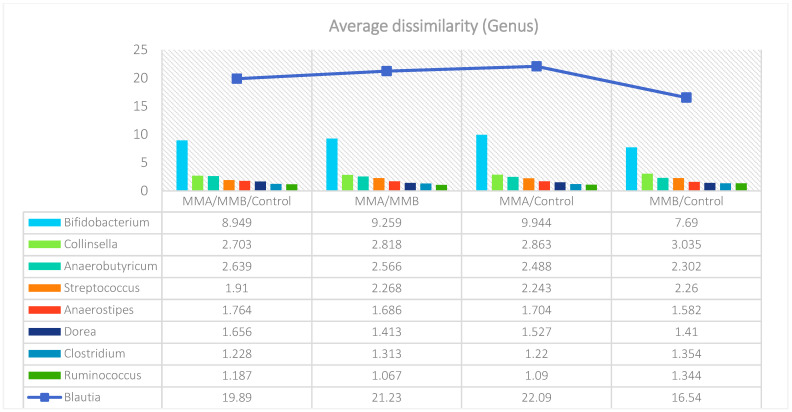
Mean divergence between the study groups (MMA—group of patients before starting the NW program; MMB—group of patients after completing the Nordic walking program) at the genus level.

**Figure 8:**
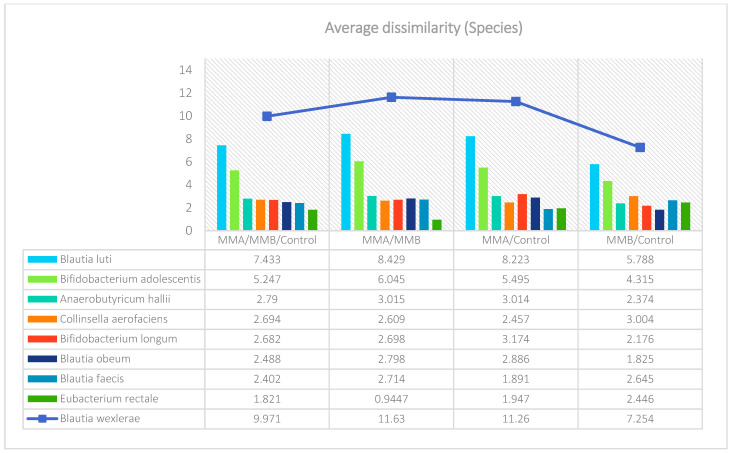
Mean divergence between the study groups (MMA—group of patients before starting the NW program; MMB—group of patients after completing the Nordic walking program) at the species level.

The analysis indicated that for the Phyllum taxon, the *Deinococcus-Thermus* and *Thermotogae* phyla were symptomatic for the MyelomaA (MMA) group at a significance level of *p* < 0.05 when comparing all groups with each other. The *Deinococcus-Thermus* phylum was also a significant taxon for MyelomaA (MMA) compared to MyelomaB (MMB). Furthermore, the *Armatimonadetes*, *Nitrospirae*, *Chrysiogenetes*, *Rhodothermaeota*, and *Dictyoglomi* phyla appeared only in the MyelomaA group, while *Lentisphaerae* was observed only in the MyelomaB group. It should be noted, however, that these taxa appeared in small quantities. A comparison of all study groups at the *Class* level revealed the taxonomic indicators *Verrucomicrobiae*, *Deinococci*, *Verrucomicrobiae*, and *Ardenticatenia* for the MyelomaA group. Only in this group was the presence of *Caldilineae*, *Chrysiogenetes*, *Dictyoglomia*, *Rhodothermia*, *Thermodesulfovibrionia*, *Thermomicrobia*, *Fimbriimonadia*, *Opitutae*, and *Ardenticatenia* also recorded. The *Deinococci* and *Actinomycetia* classes were indicator taxa for the MyelomaA group when compared with the control group (CG), and *Deinococci* was also significant when compared to the MyelomaB group. The *Lentisphaeria* class appeared only in the MyelomaB group, while the indicator taxa for the CG were the classes *Acidithiobacillia*, *Chitinophagia*, *Limnochordia*, *Longimicrobia*, *Thermodesulfobacteria*, and *Vicinamibacteria*, although the abundance of these classes was minimal. The mean taxon-specific divergences were estimated. The overall average dissimilarity calculated between the study groups (MMA, MMB, control) for all levels is shown in Table 3. The highest level of dissimilarity at all taxonomic levels was observed when comparing the control group and the patient group after NW training (MMA). Two *Phylum* groups, *Firmicutes* and *Actinobacteria*, accounted for the majority of the divergence between the study groups at the phylum level, while within the *Class* level, the main contributors were *Clostridia* and *Actinomyceta*. Among the families, *Lachnospiraceae* and *Bifidobacteriaceae* were responsible for the observed divergence between the groups, and among orders, *Eubacteriales* and *Bifidobacteriales* played this role. At the genus level, *Blautia* and *Bifidobacterium* were the main reason for the observed differences between the study groups, including species *Blautia wexlerae* and *luti* and *Bifidobacterium adolescentis* (Figure 3, Figure 4, Figure 5, Figure 6, Figure 7 and Figure 8).

**Figure 9:**
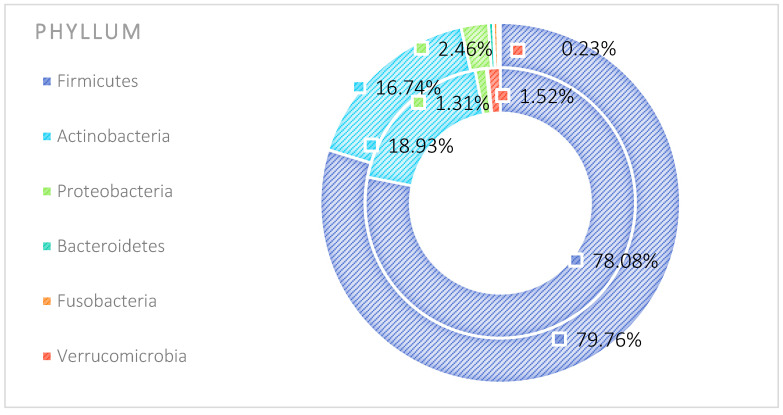
Average abundance of taxa for phyla group MMA and MMB expressed as percentage (outer circle—MMB; inner circle—MMA). MMA—group of patients before starting the NW program; MMB—group of patients after completing the Nordic walking program.

**Figure 10:**
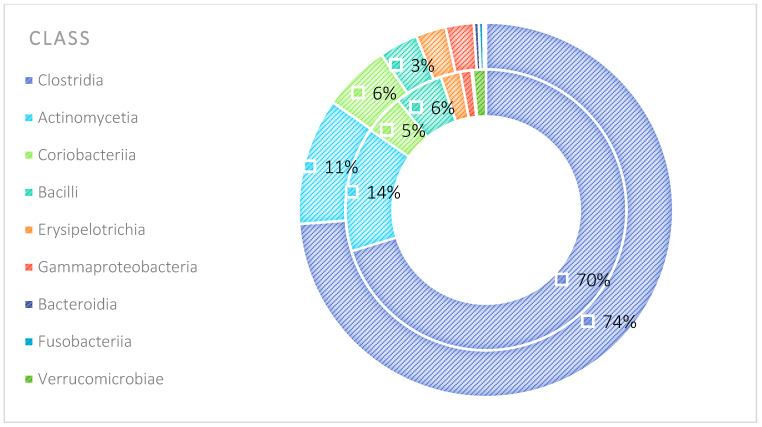
Average abundance for classes group MMA and MMB expressed as percentage (outer circle—MMB; inner circle—MMA). MMA—group of patients before starting the NW program; MMB—group of patients after completing the Nordic walking program.

**Figure 11:**
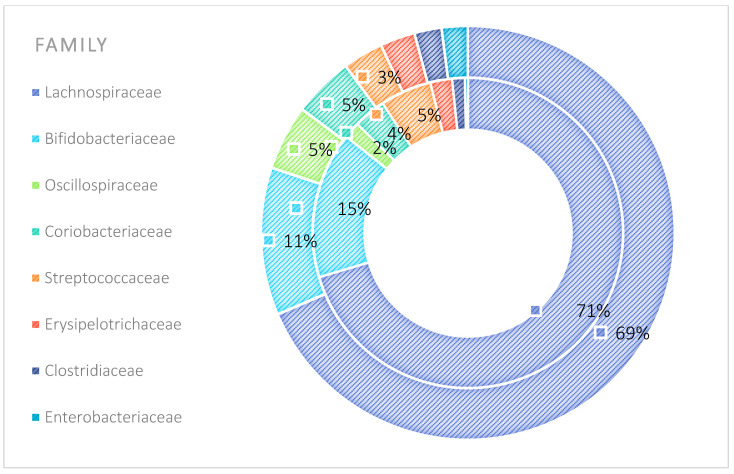
Average abundance for families group MMA and MMB expressed as percentage (outer circle—MMB; inner circle—MMA). MMA—group of patients before starting the NW program; MMB—group of patients after completing the Nordic walking program.

**Figure 12:**
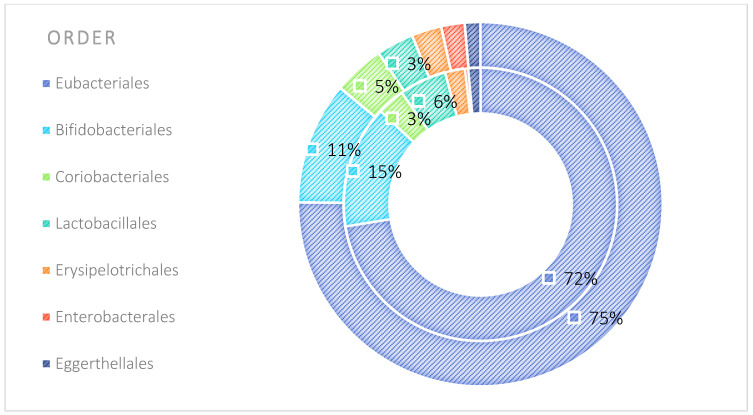
Average abundance for orders group MMA and MMB expressed as percentage (outer circle—MMB; inner circle—MMA). MMA—group of patients before starting the NW program; MMB—group of patients after completing the Nordic walking program.

**Figure 13:**
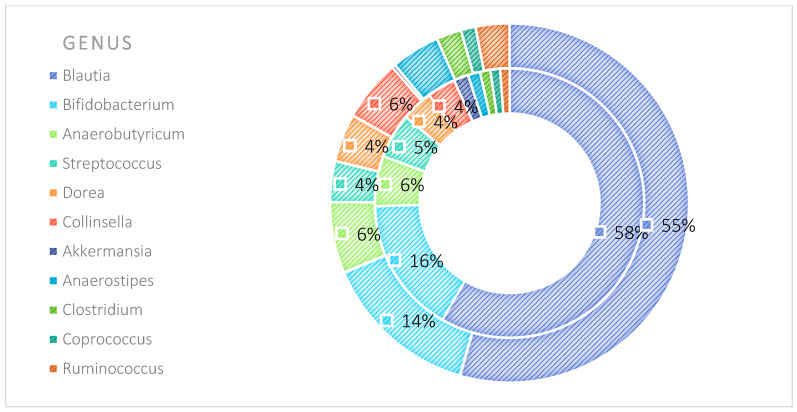
Average abundance within genus for group MMA and MMB expressed as percentage (outer circle—MMB; inner circle—MMA). MMA—group of patients before starting the NW program; MMB—group of patients after completing the Nordic walking program.

**Figure 14:**
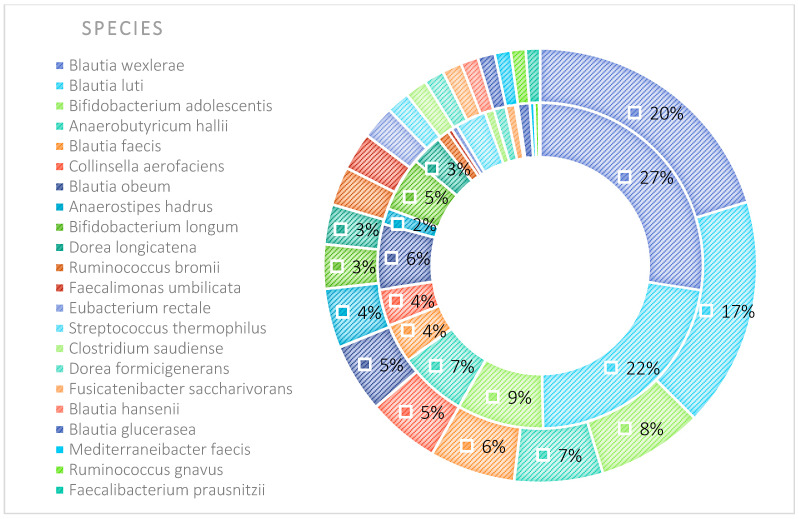
Average abundance for species group MMA and MMB expressed as percentage (outer circle—MMB; inner circle—MMA). MMA—group of patients before starting the NW program; MMB—group of patients after completing the Nordic walking program.

The microbiota of patients before starting the training was most notably represented by taxa classified into the phyla *Firmicutes*, *Actinobacteria*, *Verrucomicrobia*, *Proteobacteria*, and *Bacteroidetes* (Figure 9). After the 6-week training period combined with TRE, a rearrangement of the microbiota composition occurred. An increase in the abundance of taxa from the phyla *Proteobacteria*, *Bacteroidetes*, and *Actinobacteria* was observed. Additionally, the population of microorganisms classified into the phylum *Fusobacteria* was noted in the MyelomaB patient group. Interestingly, a decrease in the population of bacteria from the phylum *Verrucomicrobia* was observed. The population of *Firmicutes* in the studied microbiota samples showed similar abundance values both before and after the training–dietary intervention cycle. Actinobacteria showed a decrease in abundance after the training period. In this phylum, the increase mainly concerned the class *Coriobacteriia*, while a decrease was noted among the *Actinomycetia* class taxa (Figure 10). Taxa most abundantly represented after the training period compared to the pre-training period belonged to the classes *Clostridia*, *Negativicutes*, *Erysipelotrichia* (phylum *Firmicutes*), *Coriobacteria*, *Nitriliruptoria*, *Rubrobacteria* (phylum *Actinobacteria*), *Gammaproteobacteria* (phylum *Proteobacteria*), *Coriobacteria*, *Blastocatellia*, *Acidimicrobiia* (phylum *Actinobacteria*), *Bacteroidia*, and *Cytophagia* (phylum *Bacteroidetes*) (Figure 9 and Figure 10). At the family level, the rearrangement of microbiota stimulated by physical activity concerned *Lachnospiraceae* and *Bifidobacteriaceae* (Figure 11). The average abundance for each taxonomic level for the MMA and MMB groups expressed as a percentage is shown in Figure 9, Figure 10, Figure 11, Figure 12, Figure 13 and Figure 14.

**Figure 15:**
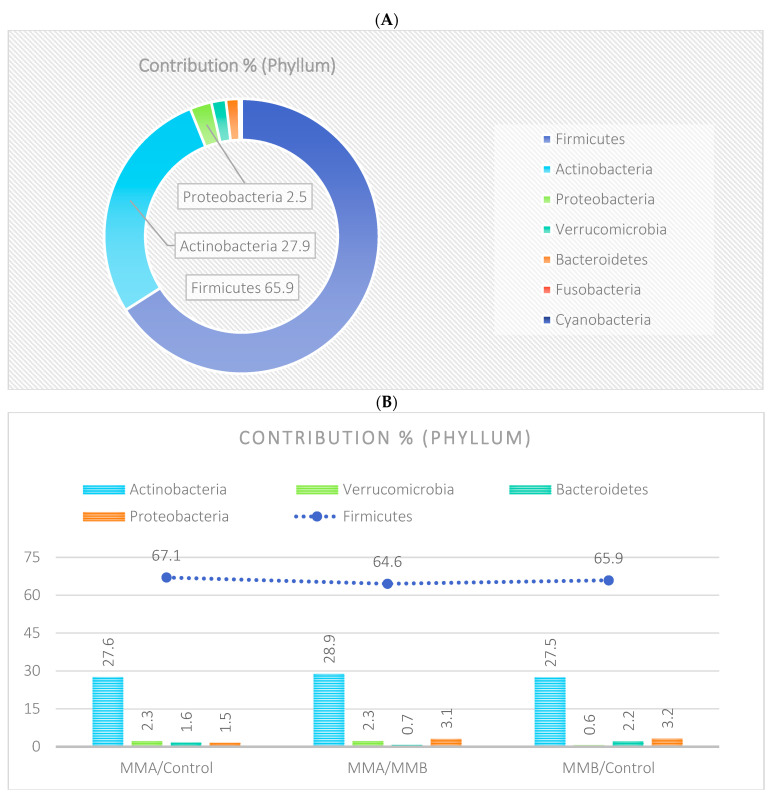

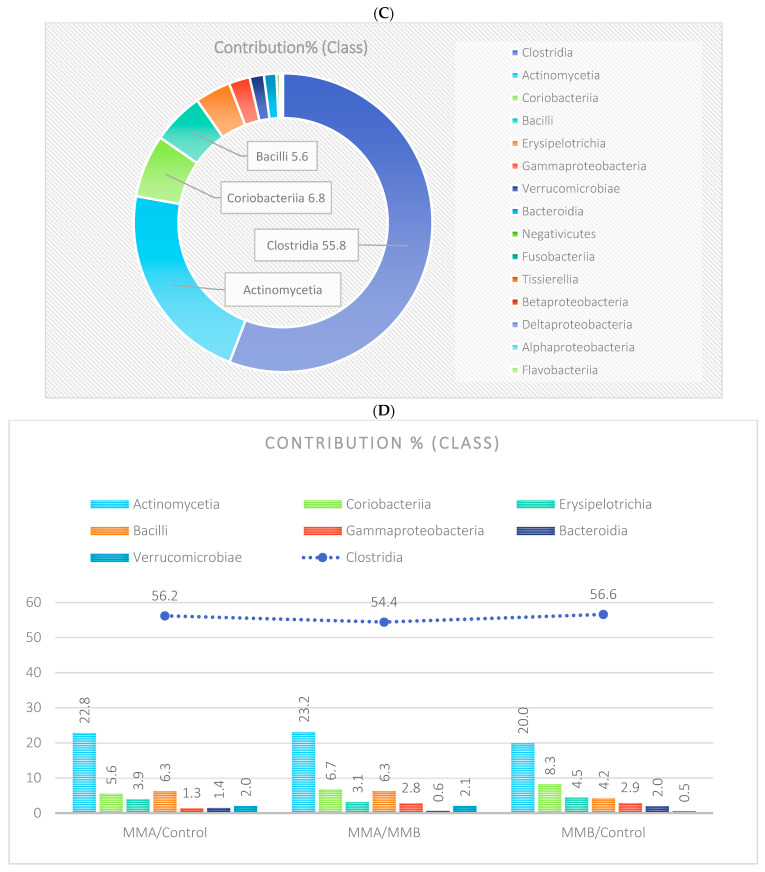

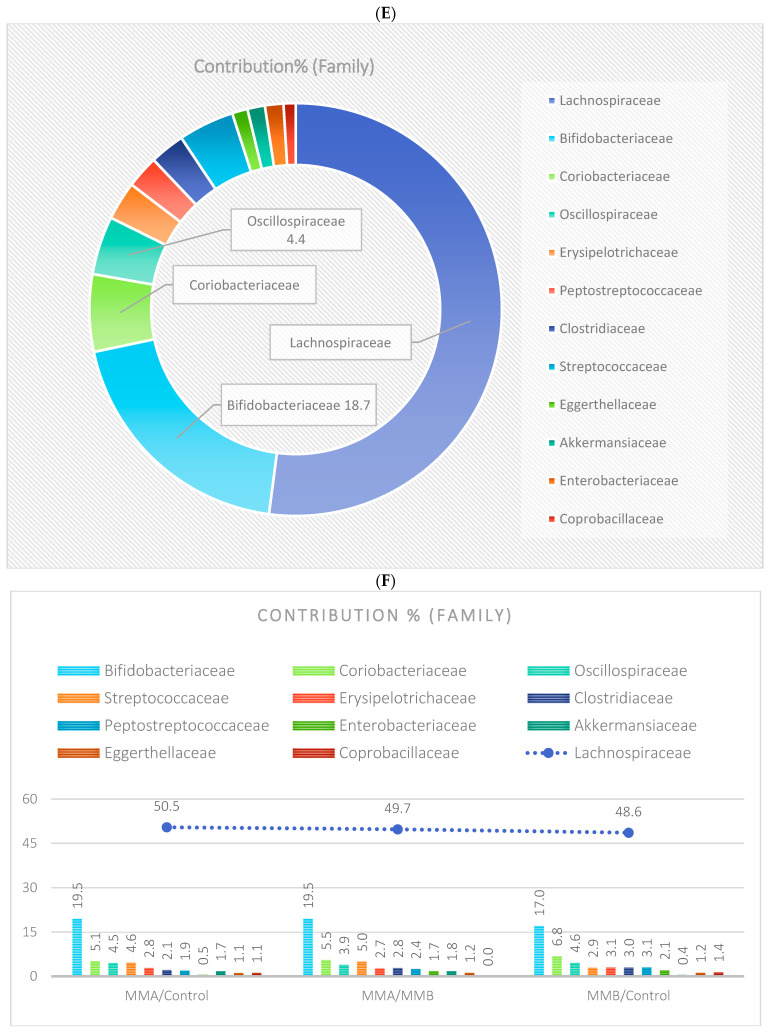

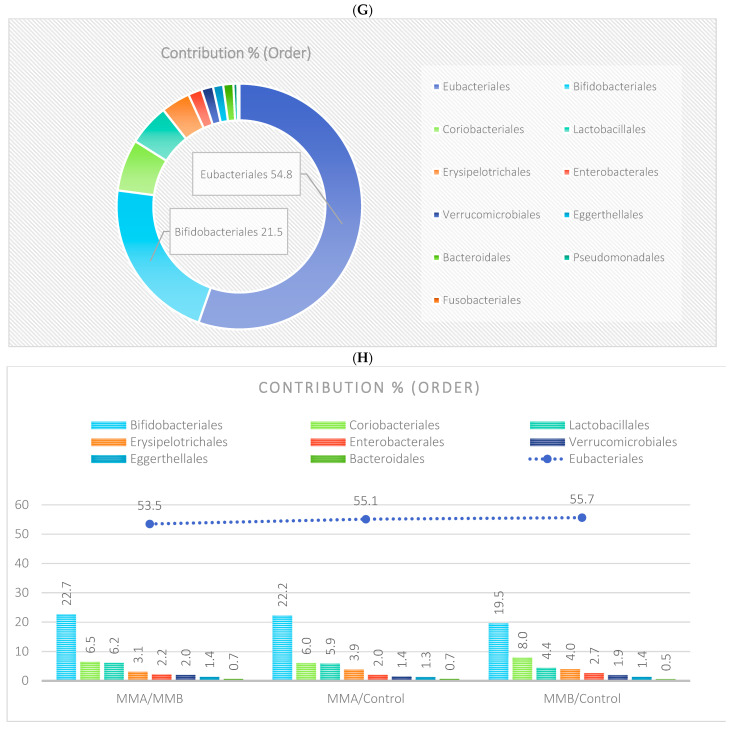

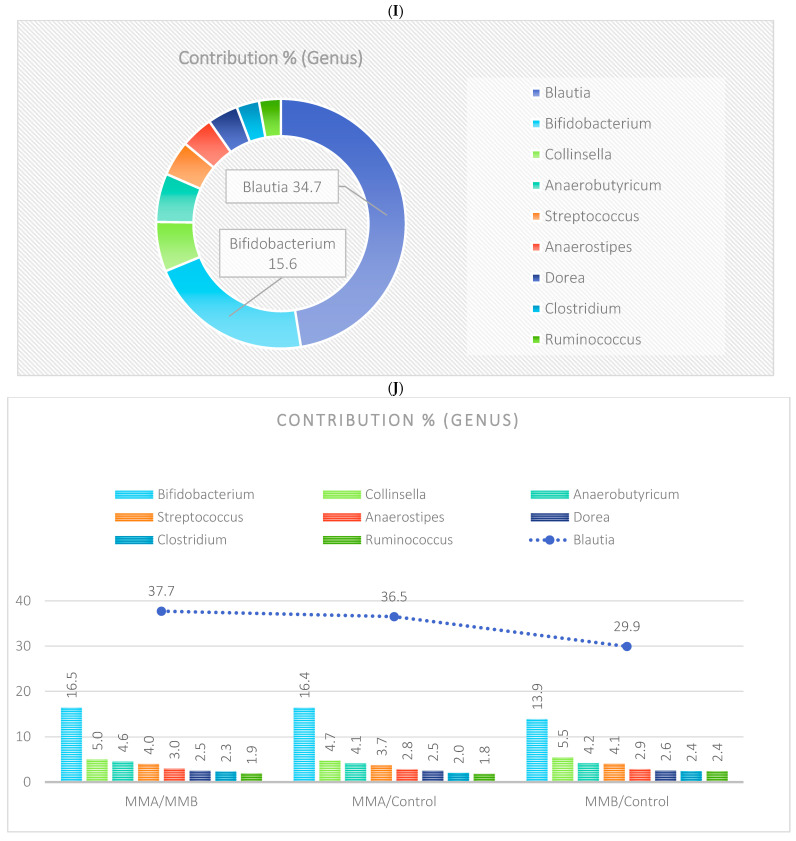

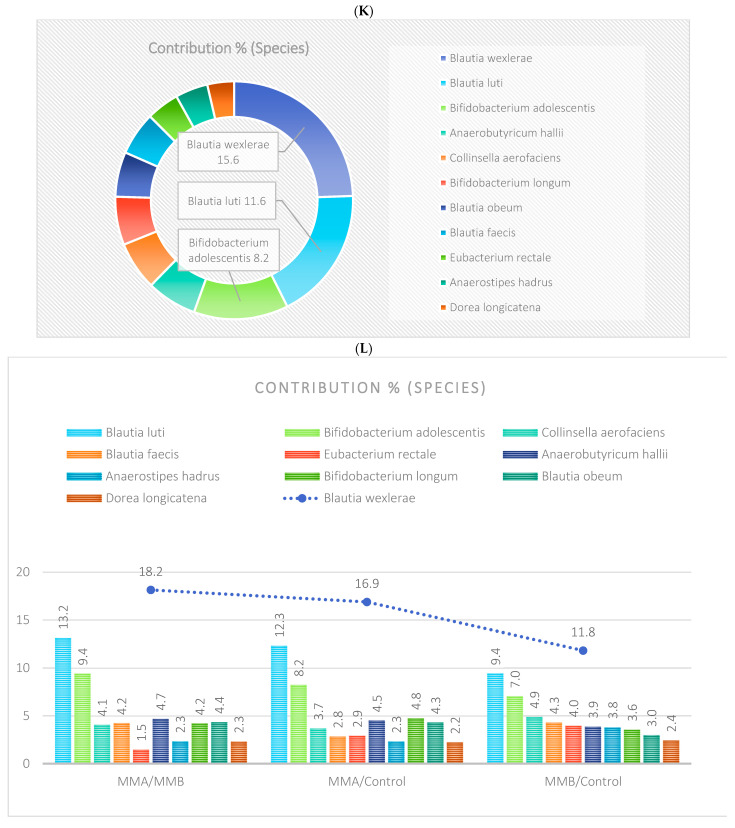
The percentage contribution of taxa within the taxonomic ranks (Phylum, Class, Family, Order, Genus, Species) with respect to the group divisions: MMA, MMB, Control. Phylum level based on the microbiota analysis of all studied samples (**A**); phylum level based on the microbiota analysis of the studied groups: MMA/MMB, MMA/Control, MMB/Control (**B**). Class level based on the microbiota analysis of all studied samples (**C**); class level based on the microbiota analysis of the studied groups: MMA/MMB, MMA/Control, MMB/Control (**D**). Family level based on the microbiota analysis of all studied samples (**E**); family level based on the microbiota analysis of the studied groups: MMA/MMB, MMA/Control, MMB/Control (**F**). Order level based on the microbiota analysis of all studied samples (**G**); order level based on the microbiota analysis of the studied groups: MMA/MMB, MMA/Control, MMB/Control (**H**). Genus level based on the microbiota analysis of all studied samples (**I**); genus level based on the microbiota analysis of the studied groups: MMA/MMB, MMA/Control, MMB/Control (**J**). Species level based on the microbiota analysis of all studied samples (**K**); species level based on the microbiota analysis of the studied groups: MMA/MMB, MMA/Control, MMB/Control (**L**).

The percentage distribution of taxa within the phylum level, calculated for all studied groups, was dominated by the phyla *Firmicutes* (66%) and *Actinobacteria* (28%). At the class level, the dominant taxa were *Clostridia*, *Actinomycetia*, *Coriobacteriia*, and *Bacilli*, together accounting for over 90% of all taxa in the microbiota of the studied sample. At the family level, the dominant taxa were *Lachnospiraceae*, *Bifidobacteriaceae*, and *Coriobacteriaceae*. At the order level, the *Eubacteriales* and *Bifidobacteriels* groups dominated, while within the genus, *Blautia* and *Bifidobacterium* had the largest contribution. In turn, the prevalent species were *Blautia wexleare* and *Blautia luti* with *Bifidonacterium adolescent* (Figure 15).

The analysis of the microbiota composition in the group of patients with multiple myeloma indicates an abundance of bacteria belonging to the families *Lachnospiraceae*, *Bifidobacteriaceae*, *Streptococcaceae*, *Clostridiaceae*, and *Erysipelotrichaceae*. After 6 weeks of NW training, the gut microbiota composition in this group of patients changed both quantitatively and qualitatively. Decrease was observed in the abundance of the families *Streptococcaceae* and *Erysipelotrichaceae*. The family *Akkermansiaceae* showed a decrease, while *Enterobacteriaceae*, *Coriobacteriaceae*, and *Peptostreptococcaceae* increased. Bacteria classified into *Fusobacteriaceae* were detected in the group of patients after the training cycle.

A comparison of the CG and the patient group MM before (MMA) and after the implementation of the training and dietary program (MMB) revealed significant differences at the Genus and Species levels of the studied microbiota (F, *p* < 0.05) (Table 4). Statistically significant changes were also observed in the microbiota of the patient group before the implementation of the NW program (MMA) and the control group again at the genus and species levels. A similar pattern was observed when comparing the control group with the MMB patient group, with no significant differences at all levels (Table 4).

**Figure 16:**
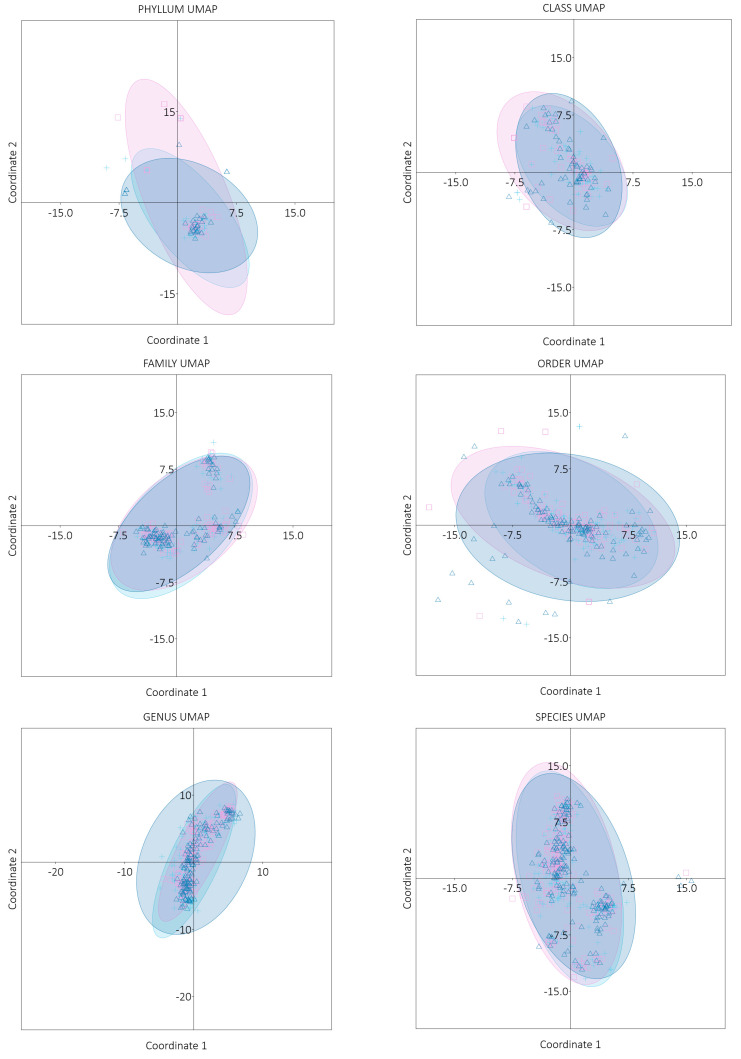
UMAP analysis performed based on Bray–Curtis metric for particular taxonomic ranks (MMA—blue plus; MMB—pink square; Control—violet triangle). The area where 95% of the population points are expected to be found (95% ellipses) was circled in the same colors. (MMA—group of patients before starting the NW program; MMB—group of patients after completing the Nordic walking program).

The Bray–Curtis metric was also used as the data matrix in the UMAP analysis. Each point on the graph represents a sample of the microbiome. The distance between points on the graph reflects the mutual similarity between microbiome samples—the closer the points are to each other, the more similar the samples are, and the further apart they are, the more different they are. The observed grouping of samples on the graph may suggest common patterns (Figure 16).

## 4. Discussion

The richness of microorganisms inhabiting the gut is influenced by many factors originating from the external environment (e.g., diet, physical activity) [5,26] as well as internal factors. A significant impact on its composition is the health status [27], which can include cancer-related diseases [12,28]. Zhang et al. [16] reported that in patients with multiple myeloma, the microbiota is most abundantly represented by taxa belonging to the phyla *Bacteroidetes* and *Firmicutes*. The results of our study confirm the presence of *Firmicutes*, but *Bacteroidetes* constituted only 1.59% to 2.44% of the total bacteria. For *Actinobacteria*, represented in the cited study at less than 1% [16], our group of patients showed approximately 62% of the total population. Pepeljugoski et al. (2019) reported that *Odoribacter* and *Lactobacillus* are genera represented in MM patients, while *Blautia* and *Faecalibacterium* are less represented in the microbiota compared to the microbiota of healthy individuals [11]. Pinart et al., in a systematic review of the microbiota in individuals with obesity, indicates a high abundance of bacteria within the *Firmicutes*. Our study reports a high abundance of bacteria in this group, which may therefore suggest a similarity in the intestinal environment as well as a similarity in the physiological functions performed by these microbes in both obese and diseased MM populations.

The results presented in this study suggest that physical activity (NW training) combined with TRE, to which the patient group was subjected, can stimulate a rearrangement of the microbiota composition toward a microbiome profile found in healthy individuals. The amount of *Faecalibacterium* increased more than tenfold (from 0.32% to 4.70%) whereas that of *Prevotella* increased from 0.03% to 0.39%.

A characteristic feature of the microbiota composition in individuals suffering from hematological diseases, including MM, is the high prevalence of bacteria from the *Firmicutes* phylum and low levels of *Proteobacteria* [29]. Our study on the microbiota composition of MM patients confirms these earlier observations. Pianko et al. [30] highlighted a correlation between this bacterium and the occurrence of minimal residual disease (MRD)—individuals with MRD had a higher abundance of bacteria from the genus *Eubacterium*. Additionally, Peled et al. [31] indicated that a higher abundance of Eubacterium in a patient’s microbiome is associated with a lower risk of MM relapse after alloHSCT. Thus, the observed more than fivefold increase in the amount of *Eubacterium* bacteria in the group of patients subjected to NW + TRE training is noteworthy. Further studies may confirm the positive, health-promoting effects of combining training and fasting.

In our previous studies, we indicated that the form of exercise in the form of NW training is safe for patients with MM. The average intensity of the NW training we adopted was based on previously obtained results, suggesting that it has a beneficial effect on blood parameters related to oxidative damage of macromolecules and gene expression associated with iron metabolism [32], and it may contribute to favorable, clinically significant changes in indicators such as the concentration of 25-OH-D vitamin [33]. There are also reports that high-intensity exercise may increase inflammation in the gut and lead to an increase in the number of associated bacteria [34,35], which would be undesirable in patients with multiple myeloma. In the microbiota of the studied group of multiple myeloma patients, after completing 6 weeks of NW training, a 15-fold increase in the number of *Prevotella* genus bacteria and a 4-fold increase in *Bifidobacterium adolescentis* were observed, which may suggest, as Humińska-Lisowska et al. [10] indicated, an improvement in performance indicators, mainly VO_2_max, in the studied group. The negative correlation of VO_2_max, estimated by Humińska-Lisowska et al. for *Bacteroides*, may also indicate the stimulation of endurance in the studied group of MM patients, as the training + TRE reduced the population of this genus by one third. Results from other studies on a similar age group of healthy individuals, where moderate-intensity exercise was applied, have been, however, ambiguous. In the study by Morita et al. [36], contrary to our findings, moderate-intensity aerobic walking training conducted for 12 weeks with a group of women over 65 years of age led to an increase in the amount of Bacteroides. In the study by Erlandson et al. [37] involving individuals aged 50–75 years, a 24-week cardiovascular and resistance training program led to an increase in *Bifidobacterium*, which was consistent with the results presented in this study, but it also reduced the amount of *Prevotella*, contrary to our findings. However, it is important to note that in the aforementioned studies, different training forms were used, and the duration of the intervention was longer than in our study. The main reason for the observed differences in post-training effects between our results and those of the cited studies is the fact that the baseline microbiota of healthy individuals differs from that of patients, which may also contribute to differences in outcomes. Therefore, future projects should focus on how training series of different durations affect the microbiome.

Many factors related to diet influence the composition of the gastrointestinal microbiota. The popularized form of dietary modification in the form of TRE is also a recognized factor in this regard [38]. Despite the lack of change in the quality of consumed food products, the change consisting of maintaining a shortened feeding window influences both the quality and quantity of the microbiome. A review by Perez-Gerdel [39] reported that changes induced by different fasting schemes positively affect the diversity and abundance of the gut microbiota. This has a direct effect on hormonal signaling, the circadian rhythm, a variety of metabolic processes, the neuronal response and immune–inflammatory pathways [18].

Other authors, however, suggest that after reviewing the available literature, we still do not have a complete picture of the impact of TRE on the microbiome. The results gathered and analyzed by Paukkonen et al. [40] were heterogeneous, and the bacteria on which intermittent fasting (IF) had a statistically significant impact that varied considerably depending on the study. Perez-Gerdel [39] also postulated that the available data currently pertain to short-term interventions. They also noted that some of the positive changes induced by fasting return to baseline. The addition of increased physical activity may therefore serve as a stimulus to augment the positive effects of TRE and/or as a factor prolonging the beneficial effect on the composition and quality of the microbiome.

To the best of our knowledge, the current study is the first attempt to examine the impact of combining two interventions: training and TRE on the gut microbiome. Previous studies have focused on isolated interventions or the combination of TRE with varying degrees of dietary modification. One such study was conducted by Mohr, who evaluated the impact of intermittent fasting (IF) and protein stimulation (P) [41]. In randomized controlled trials, they described the impact of IF and a calorie-restricted diet on the microbiological composition of stool and plasma metabolomic markers in women and men with overweight/obesity. The abundance of *Christensenellaceae* microorganisms and metabolites of amino acids promoting fat oxidation increased with IF-P.

## 5. Conclusions

The applied training intervention, combined with TRE, stimulated an increase in microbiota biodiversity and a rearrangement of the gut microbiota toward a symbiotic microbiota.

*Firmicutes* is a bacterial phylum group that is abundantly and stably represented in the gut microbiota of MM patients. Its presence was observed in patients both before starting the NW + TRE training program and after its completion, which may confirm the association of this group with the disease and indicate its dysbiotic nature. The positive effect of the 6-week NW training and fasting on the microbiota of individuals with MM, as observed in this pilot study, suggests the need for further exploration of this topic. Continued observations regarding, among other factors, different types of activities, exercise intensity, training cycle duration, and feeding window length would help optimize the program and training recommendations for patients with multiple myeloma. The observed rearrangement of the microbiota composition in MM patients toward a symbiotic microbiota appears to be an important element in supporting disease remission. And although the test is relatively expensive, learning about the microbiome can benefit many aspects of a patient’s functioning. On the other hand, learning about easy and safe tools to improve the microbiome allows us to promote them to MM patients.

## Figures and Tables

**Table 1:** Basic characteristics of selected groups: multiple myeloma group (MM) and comparison group (CG).

	Group	Mean ± SD	Independent Samples *t*-Test
BH [cm]	CG	165.56 ± 9.02	0.497
	MM	163.27 ± 11.16	
BM [kg]	CG	81.36 ± 9.09	0.598
	MM	80.24 ± 13.31	
LBM [kg]	CG	49.79 ± 5.51	0.734
	MM	51.72 ± 10.36	
SLM [kg]	CG	45.25 ± 5.02	0.651
	MM	47.42 ± 9.78	
TBW [%]	CG	44.32 ± 1.29	0.270
	MM	46.19 ± 4.62	
BMI [kg/m^2^]	CG	29.64 ± 2.60	0.836
	MM	30.07 ± 3.54	
Body fat [%]	CG	38.89 ± 2.03	<0.001 *
	MM	30.84 ± 3.27	
Age [years]	CG	62.19 ± 5.40	0.070
	MM	65.00 ± 5.13	

BH—body height; BM—body mass; LBM—lean body mass; SLM—soft lean mass; TBW—total body water. BMI—body mass index; *—statistically significant value.

**Table 2:** Assessment of the significance of differences between the studied groups (MMA—group of patients before starting the NW program; MMB—group of patients after completing the Nordic walking program; CG—control group).

Alpha Diversity				
Kruskal–Wallis Test for Equal Medians (*p*)		Mann–Whitney Pairwise (*p*)		
Indices		MMA-MMB	MMA-CG	MMB-CG
Species				
Simpson_1-D	0.004 *	0.300	0.002 *	0.519
Shannon_H	0.002 *	0.378	0.001 *	0.428
Fisher_alpha	0.031 *	1.000	0.050 *	0.127
Genus				
Simpson_1-D	0.012 *	0.472	0.006 *	0.948
Shannon_H	0.005 *	0.378	0.003 *	0.428
Fisher_alpha	0.012 *	0.472	0.006 *	0.948
Order				
Simpson_1-D	0.791	1.000	1.000	1.000
Shannon_H	0.908	1.000	1.000	1.000
Fisher_alpha	0.820	1.000	1.000	1.000
Family				
Simpson_1-D	0.564	1.000	0.806	1.000
Shannon_H	0.262	1.000	0.255	1.000
Fisher_alpha	0.343	1.000	0.472	1.000
Class				
Simpson_1-D	0.765	1.000	1.000	1.000
Shannon_H	0.851	1.000	1.000	1.000
Fisher_alpha	0.716	1.000	1.000	1.000
Phyllum				
Simpson_1-D	0.959	1.000	1.000	1.000
Shannon_H	0.859	1.000	1.000	1.000
Fisher_alpha	0.146	1.000	1.000	0.112

*—statistically significant value.

**Table 3:** Overall average dissimilarity calculated between the study groups using the Bray–Curtis measure (MMA—group of patients before starting the NW program; MMB—group of patients after completing the Nordic walking program; CG—control group) for all levels. The *p*-values were assessed with ANOSIM.

Overall Average Dissimilarity	MMA/MMB/CG	MMA/MMB	MMA/CG	MMB/CG
Species	64.03	64.11	66.67	61.31
*p*	0.009 *	0.65	0.0003 *	0.05 *
Genus	57.39	56.26	60.49	55.27
*p*	0.02 *	0.53	0.0008 *	0.11
Order	39.69	39.36	42.61	37.04
*p*	0.1897	0.623	0.04 *	0.4709
Family	39.69	39.36	42.61	37.04
*p*	0.094	0.55	0.01 *	0.46 *
Class	38.94	38.65	41.73	36.38
*p*	0.21	0.61	0.05 *	0.52
Phyllum	34.97	34.82	37.47	32.61
*p*	0.31	0.74	0.08	0.51

*—statistically significant value.

**Table 4:** Bray–Curtis dissimilarity between the studied groups (PerMANOVA test). MMA—group of patients before starting the NW program; MMB—group of patients after completing the Nordic walking program; CG—control group.

Bray–Curtis Distance					
PERMANOVA	F	p	MMA-MMB	MMA-CG	MMB-CG
Phyllum	1.21	0.30	1.00	0.32	0.97
Class	1.37	0.22	1.00	0.22	0.88
Family	1.69	0.12	1.00	0.07	0.97
Order	1.45	0.20	1.00	0.21	0.81
Genus	2.19	0.03 *	1.00	0.0057 *	0.35
Species	1.93	0.03 *	1.00	0.0057 *	0.24

*—statistically significant value.

## Data Availability

The data presented in this study are available on request from the corresponding authors.
